# Supplementary material for: A framework for detecting and predicting highway traffic anomalies via multimodal fusion and heterogeneous graph neural networks
Source: PLoS One. 2025 Jun 25;20(6):e0326313. doi: 10.1371/journal.pone.0326313 (PMC12193924; doi:10.1371/journal.pone.0326313)
Supplement: Attachment — Submitted filename: Response.docx [file pone.0326313.s002.docx]

Dear Editorial Department of PLOS ONE:

We would like to thank you for your review of our paper "A framework for detecting and predicting abnormal traffic events on highways based on multimodal deep fusion and heterogeneous graph neural networks: a method of integrating and comparing pessimistic likelihood estimation algorithms" and for your valuable review comments. We have carefully read and considered each review comment, and have made changes to the paper, and the changes have been highlighted. Below are our point-by-point responses to the review comments:

Journal requirements:

When submitting your revision, we need you to address these additional requirements.

1. Please ensure that your manuscript meets PLOS ONE's style requirements, including those for file naming. The PLOS ONE style templates can be found at

https://journals.plos.org/plosone/s/file?id=wjVg/PLOSOne_formatting_sample_main_body.pdf and

<https://journals.plos.org/plosone/s/file?id=ba62/PLOSOne_formatting_sample_title_authors_affiliations.pdf>

**Response:** Thank you for your reminder. We have carefully reviewed and updated our manuscript to ensure it meets the PLOS ONE style requirements. We have also double-checked the file naming conventions and made the necessary adjustments. If there are any further specific requirements or adjustments needed, please let us know.

2. Please note that PLOS ONE has specific guidelines on code sharing for submissions in which author-generated code underpins the findings in the manuscript. In these cases, we expect all author-generated code to be made available without restrictions upon publication of the work. Please review our guidelines at https://journals.plos.org/plosone/s/materials-and-software-sharing#loc-sharing-code and ensure that your code is shared in a way that follows best practice and facilitates reproducibility and reuse.

**Response:** Thank you for your reminder. We have reviewed the PLOS ONE guidelines on code sharing and have made the author-generated code publicly available via a GitHub repository. The code can be accessed using the following link: https://github.com/ShaoweiSun/A-framework-for-detecting-and-predicting-highway-traffic-anomalies-.git. We have also included the necessary documentation and licensing information to facilitate reproducibility and reuse.

3. We note that the grant information you provided in the ‘Funding Information’ and ‘Financial Disclosure’ sections do not match. When you resubmit, please ensure that you provide the correct grant numbers for the awards you received for your study in the ‘Funding Information’ section.

**Response:** Thank you for bringing this to our attention. We have reviewed the funding information and ensured that the correct grant numbers are provided in the ‘Funding Information’ section. The grant number for the study is 2022-YF044 from the Science and Technology Project of Housing and Urban-Rural Construction of Anhui Province.

4. Thank you for stating the following in the Acknowledgments Section of your manuscript:

“The authors would like to express their sincere gratitude to the Science and Technology Project of Housing and Urban-Rural Construction of Anhui Province (Grant No. 2022-YF044) for their financial support, which enabled the research presented in this paper. We are also grateful to the reviewers for their constructive comments and suggestions, which helped improve the quality of this manuscript.

Special thanks go to the Intelligent Transportation Engineering Institute and the School of Mechanical Engineering at Hefei University of Technology for providing the necessary research facilities and resources. We appreciate the contributions of our colleagues and research assistants for their valuable insights and support throughout the project.

The authors would also like to acknowledge the data providers and contributors of the publicly available traffic datasets used in this study. Their efforts in collecting and sharing such valuable data have significantly enhanced the comprehensiveness and reliability of our research findings.

Lastly, we extend our appreciation to our families for their unwavering support and encouragement, which have been instrumental in the successful completion of this work.”

We note that you have provided funding information that is not currently declared in your Funding Statement. However, funding information should not appear in the Acknowledgments section or other areas of your manuscript. We will only publish funding information present in the Funding Statement section of the online submission form.

Please remove any funding-related text from the manuscript and let us know how you would like to update your Funding Statement. Currently, your Funding Statement reads as follows:

“The author(s) received no specific funding for this work.”

Please include your amended statements within your cover letter; we will change the online submission form on your behalf.

**Response:** Thank you for your guidance. We have removed the funding-related text from the Acknowledgments section. Please update the Funding Statement to read: "This work was supported by the Science and Technology Project of Housing and Urban-Rural Construction of Anhui Province, Grant No. 2022-YF044."

5. We note that your Data Availability Statement is currently as follows: [All relevant data are within the manuscript and its Supporting Information files.]

Please confirm at this time whether or not your submission contains all raw data required to replicate the results of your study. Authors must share the “minimal data set” for their submission. PLOS defines the minimal data set to consist of the data required to replicate all study findings reported in the article, as well as related metadata and methods (https://journals.plos.org/plosone/s/data-availability#loc-minimal-data-set-definition).

For example, authors should submit the following data:

- The values behind the means, standard deviations and other measures reported;

- The values used to build graphs;

- The points extracted from images for analysis.

Authors do not need to submit their entire data set if only a portion of the data was used in the reported study.

If your submission does not contain these data, please either upload them as Supporting Information files or deposit them to a stable, public repository and provide us with the relevant URLs, DOIs, or accession numbers. For a list of recommended repositories, please see https://journals.plos.org/plosone/s/recommended-repositories.

If there are ethical or legal restrictions on sharing a de-identified data set, please explain them in detail (e.g., data contain potentially sensitive information, data are owned by a third-party organization, etc.) and who has imposed them (e.g., an ethics committee). Please also provide contact information for a data access committee, ethics committee, or other institutional body to which data requests may be sent. If data are owned by a third party, please indicate how others may request data access.

**Response:** Thank you for your guidance. We confirm that our submission contains all the raw data required to replicate the study's results. The minimal data set, including the values behind the means, standard deviations, and graph - building values, is available within the manuscript and its Supporting Information files. We have also ensured compliance with PLOS ONE's definition of the minimal data.

**Reviewer #1:** Suggest:

This paper addresses the problem that traditional traffic detection models usually rely on single data sources. The authors propose a new framework for detecting and predicting abnormal traffic events on highways. The method uses multimodal deep fusion and heterogeneous graph neural networks to effectively fuse multi-source heterogeneous traffic data. It also applies the CPLE algorithm to improve performance. As a result, the accuracy of real-time abnormal event detection is improved. The research is novel and meaningful, especially for handling heterogeneous traffic data. The experiments are also rich and detailed. However, the current version still does not meet the journal's standards. Some problems need to be fixed. The main issues are as follows:

1. The title is too long. Please make it shorter and focus on the key point of the research.
**Response:** Thank you for your valuable feedback regarding the title of our manuscript. We agree that the original title was quite lengthy and could benefit from simplification to better emphasize the core of our research. Based on your suggestion, we have revised the title to "A Framework for Detecting and Predicting Highway Traffic Anomalies via Multimodal Fusion and Heterogeneous Graph Neural Networks." The modified content is as follows:

**A Framework for Detecting and Predicting Highway Traffic Anomalies via Multimodal Fusion and Heterogeneous Graph Neural Networks**

2. The abstract is also too long. It only needs to briefly mention the research background, main idea, and general experimental results. There is no need to list detailed results like accuracy or F1 scores of different models.

**Response:** Thanks for your feedback. We've revised the abstract to focus on the research background, main idea, and general results, removing specific metrics. The modified content is as follows:

**Abstract**

This paper presents a novel framework for detecting and predicting abnormal traffic events on highways. Current traffic monitoring systems often rely on single data sources, which limits their detection accuracy and robustness in complex environments. To address these limitations, we propose a framework based on multimodal deep fusion and heterogeneous graph neural networks (HGNNs), incorporating an Ensemble Contrastive Pessimistic Likelihood Estimation (CPLE) algorithm to optimize performance. The framework integrates static and dynamic traffic data, such as video images, traffic flow, vehicle speed, and tunnel weather conditions. Experimental results demonstrate that the model performs well in various scenarios, showing significant improvement in accuracy and stability over existing models like AGC-LSTM and AttentionDeepST. For instance, the proposed MHGNN-CPLE model achieves superior accuracy and F1 score in static detection tasks while maintaining high accuracy under different noise levels in dynamic detection scenarios. This study provides a significant advancement in traffic event analysis by effectively combining multimodal data and leveraging HGNNs to capture complex spatiotemporal dependencies.

3. The logic in the section Introduction is not very clear. It is better to first introduce the research background, then review existing work and point out its limitations. After that, present your own model. This will make the structure more logical. At the end of the introduction, please summarize the main contributions so readers can understand the paper more easily.

**Response:** Thank you for your feedback on the introduction section. We have revised the introduction to first present the research background, followed by a review of existing work and its limitations, and then introduce our proposed model. The main contributions are summarized at the end of the introduction. This new structure should provide a clearer and more logical flow for readers.

4. In the related work section, it would be helpful to add a table comparing existing highway abnormal event detection models with the proposed model. This will better show the advantages of your method.
**Response:** Thank you for your suggestion. We have revised the "Related Work" section to include a comparative table that highlights the differences between existing models for highway abnormal event detection and our proposed model. This table aims to clearly demonstrate the unique features and advantages of our approach.

5. Figure 1 shows the model framework, which includes data collection and fusion, model development and evaluation, and model optimization. But the beginning of **Response:** Section 3 does not match this structure. Please check this part and revise it carefully. Thank you for pointing out the inconsistency between the beginning of Section 3 and Figure 1. We have carefully revised Section 3 to align with the model framework presented in Figure 1. The modified content is as follows:

In this research, a framework for detecting and predicting abnormal traffic events on highways based on multimodal deep fusion and heterogeneous graph neural networks (HGNNs) is constructed. The aim is to overcome the limitations of traditional methods and enhance the accuracy and robustness of detection and prediction. This framework mainly encompasses three core components: data collection and fusion, model development and evaluation, and model optimization. The overall architecture is shown in Figure 1.

6. There are some errors in lines 175–176, 183–185, 193–195, and 198–199. Some parameter letters are missing. Please correct these sentences and carefully check the whole text to correct spelling mistakes.

**Response:** Thank you for your valuable comments. We have carefully addressed the issues in lines 175–176, 183–185, 193–195, and 198–199, and corrected the missing parameter letters. The revised equations and explanations have been integrated into the manuscript without altering the original structure. We have also conducted a thorough check to ensure no other spelling mistakes are present.

7. The paper uses multimodal deep fusion and heterogeneous graph neural networks to detect and predict highway abnormal traffic events. It is suggested to add ablation experiments in the experimental section. You can compare different data source combinations to see how they affect the model's performance. You should also study how each part of the model affects the results.
**Response:** Thank you for your suggestion. We have added an ablation study section to the experimental part of our paper. This section evaluates the contributions of different data sources and model components by systematically removing them and assessing the impact on model performance. The results demonstrate the importance of multimodal data fusion and each component of our model in achieving high accuracy and robustness in detecting and predicting abnormal traffic events.

8. Related work is quite incomplete. Many related SOTA traffic prediction studies are not reviewed in this paper.
Zhang J, Mao S, Yang L, et al. Physics-informed deep learning for traffic state estimation based on the traffic flow model and computational graph method[J]. Information Fusion, 2024, 101: 101971.
Zhang S, Zhang J, Yang L, et al. Physics Guided Deep Learning-based Model for Short-term Origin-Destination Demand Prediction in Urban Rail Transit Systems Under Pandemic[J]. Engineering, 2024.
Zhang J, Mao S, Zhang S, et al. EF-former for short-term passenger Flow Prediction during large-scale events in Urban Rail Transit systems[J]. Information Fusion, 2025, 117: 102916.
Zhang J, Zhang S, Zhao H, et al. Multi-frequency spatial-temporal graph neural network for short-term metro OD demand prediction during public health emergencies[J]. Transportation, 2025: 1-23.
Qiu H, Zhang J, Yang L, et al. Spatial–temporal multi-task learning for short-term passenger inflow and outflow prediction on holidays in urban rail transit systems[J]. Transportation, 2025: 1-30.

**Response:** Thank you for pointing out the incompleteness of the related work. We have revised the related work section to incorporate the recent studies you mentioned. We briefly introduce the key contributions of these works and how they relate to our research. We have also updated the comparison table in the state-of-the-art techniques section to reflect these new additions. This enhancement provides a more comprehensive overview of the current research landscape and better positions our contribution within it.

**Reviewer #2:**

1. The manuscript should have a section to describe state-of-the-art techniques. This section should also outline a tabular sketch so that it is easy to identify what’s missing in the literature and how this paper addresses that. This section can be derived from contents described in the introduction section.

**Response:** Thank you for your suggestion. We have added a new section titled "Comparative Analysis of Existing Models" after the introduction. This section provides an overview of the latest methods in highway traffic anomaly detection and includes a table that contrasts these techniques with our proposed framework. The table highlights the limitations of existing research and demonstrates how our work addresses these gaps. The modified content is as follows:

**Comparative Analysis of Existing Models**

Recent studies have significantly advanced the field of traffic prediction and abnormal event detection by integrating various methodologies and applying cutting-edge technologies. Zhang et al. (2024) proposed a physics-informed deep learning framework that combines traffic flow models with computational graph methods, achieving improved accuracy in traffic prediction tasks [28]. Zhang et al. (2024) further extended this approach by developing a physics-guided deep learning model for short-term origin-destination demand prediction in urban rail transit systems, demonstrating robust performance under pandemic conditions [29]. Zhang et al. (2025) introduced the EF-former model for short-term passenger flow prediction during large-scale events in urban rail transit systems, leveraging the power of transformers to capture complex spatiotemporal dependencies [30]. Additionally, Zhang et al. (2025) presented a multi-frequency spatial-temporal graph neural network for short-term metro origin-destination demand prediction during public health emergencies, further advancing the application of graph neural networks in traffic prediction [31]. Qiu et al. (2025) proposed a spatial–temporal multi-task learning method for predicting passenger inflow and outflow in urban rail transit systems, particularly during holidays [32]. Sharafian et al. (2025) explored fuzzy adaptive control strategies for consensus tracking in multi-agent systems with incommensurate fractional-order dynamics, offering innovative solutions for managing traffic in uncertain and dynamic environments [24]. Sharafian et al. (2025) also investigated the resilience of adaptive RBF neural networks against deception attacks in consensus tracking control of incommensurate fractional-order power systems, highlighting the importance of robustness in intelligent transportation systems [22]. Ali et al. (2025) proposed an energy-efficient resource allocation strategy for urban traffic flow prediction in edge-cloud computing environments, optimizing resource usage while maintaining prediction accuracy [23]. Ali et al. (2024) introduced a resource-aware multi-graph neural network for urban traffic flow prediction in multi-access edge computing systems, further advancing the application of graph neural networks in traffic prediction [26]. Ali et al. (2025) presented an attention-driven spatio-temporal deep hybrid neural network for traffic flow prediction in transportation systems, effectively capturing complex spatial and temporal dependencies in traffic data [25]. Ali et al. (2021) proposed a data aggregation-based approach to exploit dynamic spatiotemporal correlations for citywide crowd flows prediction in fog computing, providing valuable insights into understanding and predicting traffic patterns in complex urban environments [27]. To better illustrate the advantages of our proposed framework, Table 1 provides a comprehensive comparison of existing highway abnormal event detection models with our method. The comparison covers various aspects, including model type, data types used, spatial-temporal dependency modeling, and performance metrics. The results highlight the superiority of our multimodal deep fusion and heterogeneous graph neural network (HGNN) framework in terms of accuracy, robustness, and adaptability to complex traffic scenarios.

2. Authors should pattern the motivation behind using this method to explain in the introduction.

**Response:** Thank you for your feedback. We have revised the introduction to clarify the motivation for using the proposed method.

3. The abstract is not coherent. It would be good if authors can write a sentence describing numerical results and improvement over other methods.

**Response:** Thank you for your feedback. We have revised the abstract to make it more coherent and included a sentence describing the numerical results and improvements over other methods.

4. The integration of diverse data types (video, speed, flow, weather) strengthens detection accuracy and robustness. However, the fusion strategy (early, late, or hybrid fusion) should be clearly explained, including how modalities are synchronized and weighted.

**Response:** Thank you for your feedback. We have clarified the fusion strategy in the revised manuscript, explaining that we employed a hybrid fusion approach combining early and late fusion. We also detailed how modalities are synchronized through time-stamping and alignment algorithms and weighted based on historical data analysis.

5. There needs to be citation of recent papers on this topic and revise the literature section with slight Incorporation of recent ideas. Add more recent works in related section (Resilience to deception attacks in consensus tracking control of incommensurate fractional-order power systems via adaptive RBF neural network, Energy‐Efficient Resource Allocation for Urban Traffic Flow Prediction in Edge‐Cloud Computing, Fuzzy adaptive control for consensus tracking in multiagent systems with incommensurate fractional-order dynamics: Application to power systems, An Attention-Driven Spatio-Temporal Deep Hybrid Neural Networks for Traffic Flow Prediction in Transportation Systems, A resource-aware multi-graph neural network for urban traffic flow prediction in multi-access edge computing systems, A data aggregation based approach to exploit dynamic spatio-temporal correlations for citywide crowd flows prediction in fog computing)

**Response:** Thank you for your insightful comments. We have updated the literature section to incorporate recent works, including those on adaptive RBF neural networks for power systems, energy-efficient traffic flow prediction, fuzzy adaptive control, and attention-driven spatio-temporal neural networks. These additions strengthen the review of recent advancements in the field.

6. The CPLE algorithm appears to address uncertainty or imbalance in anomaly prediction, which is a common challenge. However, the intuition behind CPLE, its objective function, and how it contrasts with standard loss functions need elaboration.
Were any temporal GNNs (like ST-GCN or T-GCN) considered?

**Response:** Thank you for your valuable feedback. We have elaborated on the intuition behind the CPLE algorithm, its objective function, and its contrast with standard loss functions in the revised manuscript. The modified content is as follows:

## 1. Introduction

“...”

Our proposed framework aims to improve the accuracy and reliability of detecting and predicting abnormal traffic events. The framework effectively integrates multimodal data and leverages HGNNs to capture complex spatiotemporal dependencies. The Ensemble Contrastive Pessimistic Likelihood Estimation (CPLE) algorithm is integrated to address the common challenge of uncertainty and imbalance in anomaly prediction. Unlike standard loss functions that treat all samples uniformly, CPLE dynamically adjusts sample weights based on their similarity and prediction errors. By emphasizing the differences between samples, CPLE enhances the model's ability to recognize anomaly events. Its objective function is designed to minimize the contrastive pessimistic likelihood, thereby improving the detection accuracy of abnormal traffic events even in imbalanced datasets.

**Application of Graph Neural Networks in Traffic Prediction**

In traffic flow prediction and anomaly detection, graph neural networks (GNNs) are increasingly used to manage complex spatiotemporal data. They effectively capture spatial dependencies and their dynamic changes in traffic flow, especially in transportation networks, due to their superior information transmission capabilities [19]. Although temporal GNNs such as ST-GCN and T-GCN have shown promise in capturing temporal dependencies, our framework focuses on leveraging HGNNs to handle diverse data types and complex traffic events. However, the integration of temporal GNNs could be a valuable direction for future research.

7. Pattern the motivation behind using this method to explain in the introduction. Why the existing schemes failed? Does no study try to address this aspect before? If yes, this has to be mentioned.

**Response:** Thank you for your feedback. We have revised the introduction to clarify the motivation behind using our method. We explain why existing schemes fall short and mention the gap in previous studies regarding the comprehensive integration of multimodal data with HGNNs.

8. Parameters of network have been enhanced using training data "until the model obtains the maximum accuracy". If this accuracy is the training accuracy, maybe over-fitting has been performed. If this accuracy is the testing accuracy, the system is adjusted over the same subset that is evaluated. A validation subset could be used to optimize the system with different data than the testing data and without performing over-fitting. In addition, it would be interesting to know which range of each parameter has been analyzed."?

**Response:** Thank you for your feedback. We have revised the experiment section to clarify that a validation subset was used to optimize the model parameters, separate from the training and testing data, to avoid overfitting. We have also specified the range of parameters analyzed. The modified content is as follows:

**Experiment and result analysis**

**Dataset selection and experimental parameter setting and process**

*“...”*

The training and validation sets were designed to ensure the model could effectively learn and predict in different traffic modes. The training set included traffic flow data from the METR - LA and PEMS - BAY datasets, which were normalized and time - aligned to cover features such as vehicle speed, flow, and density for multiple road segments and different time periods. Normal and abnormal data were balanced using oversampling and undersampling methods. The validation set was randomly selected from the raw data to ensure no duplication with the training set. It was mainly used to evaluate the model's generalization ability and cover various traffic modes. The ratio of the training set to the validation set was typically 80% and 20%, ensuring the model could fully learn during training and its performance could be effectively evaluated during validation. To optimize the model parameters and avoid overfitting, we used a validation subset separate from both the training and testing data. The parameters were tuned based on the validation performance. Specifically, we analyzed a range of values for each parameter, such as learning rates from 0.0001 to 0.01, batch sizes from 32 to 128, and dropout rates from 0.3 to 0.7, to find the optimal settings that maximize the model's accuracy on the validation set.

Thank you again for your valuable feedback. We believe these revisions have significantly improved the quality and clarity of our manuscript. We look forward to the opportunity to contribute to the PLOS ONE.

Sincerely,

Shaowei Sun

School of Mechanical Engineering, Hefei University of Technology, Hefei, Anhui,China
